# Relapses in Illicit Drug Use Among Probationers: Results in a Risk Group of Public Health Services in Bavaria

**DOI:** 10.3389/ijph.2023.1605955

**Published:** 2023-10-11

**Authors:** U. Kappl, A. M. Sakr, B. Huppertz, H. Stöver, H. Stich

**Affiliations:** ^1^ Institute for Medical Information Processing, Biometry, and Epidemiology, Faculty of Medicine, Ludwig Maximilian University of Munich, Munich, Germany; ^2^ Pettenkofer School of Public Health, Munich, Germany; ^3^ Department of Toxicology and Drug Monitoring, MVZ Laboratory Dr. Quade & Colleagues GmbH, Cologne, Germany; ^4^ Frankfurt University of Applied Sciences, Frankfurt, Germany; ^5^ Department of Public Health Medicine, Landshut, Germany

**Keywords:** drugs, relapses, probation, time trends, risk population

## Abstract

**Objective:** We aimed to identify in this study time trends of relapses in the illicit consumption of narcotics in a special at-risk population of former drug users under a public health perspective.

**Methods:** In a pooled dataset of 14 consecutive calendar years (2006–2019), the use of seven different narcotic substances was studied in 380 persons with a total of 2,928 urine samples which were analyzed using a valid marker system for narcotic residues.

**Results:** During the entire observation period, the relapse rate for cannabinoids and opiates was the highest despite abstinence requirements. It was noticeable that the relapses across all narcotics groups occurred primarily during the first 3 years of the probation period (90%) with a decrease in illegal consumption during the following years of the observation period.

**Conclusion:** Special attention should be paid to probationers at the beginning of the probation period to develop more effective prevention strategies for substance abstinence by all involved actors in public health services.

## Introduction

The diverse causes and consequences of illicit drug use pose a challenge to society as a whole [1–5], which is recently characterized by a variety of new addictive substances and specific consumption patterns [1, 3–5]. Determinants for recidivism of previous narcotics users and other illicit drugs include modifiable and non-modifiable risk factors. Modifiable factors include the intensity of substance abuse and the associated need for addiction treatment. Non-modifiable risk factors include younger age, male sex, and criminal history [6–8]. However, depending on the risk constellation, the social environment could pose a health risk or have a positive impact on individuals with no record of illicit substance use [9–11].

When it comes to violations and breaches of abstinence requirements during a legally binding probation order, Jehle et al. [12] found that convictions based on the German Narcotics Act (BtMG) resulted in an above-average recidivism rate. MacKenzie et al. [13] determined a reduction in illicit narcotics use from 69% to 27% in the first probationary year. However, according to Caudy et al. [14], abstaining from the use of so-called hard drugs during a legally binding probationary period is less common, especially among young people aged 18–25 years. Regarding the timing of recidivism throughout the probationary period, Gray et al. [15] found that about 30% of subjects recidivated within the first 100 days of probation. Although these working groups [12–16] have investigated the temporal aspects of recidivism for the at-risk population while on probation, there is still a considerable knowledge gap on this topic.

This long-term study aimed to quantify the illicit narcotics use by probationers despite an absolute substance-specific abstinence court order. Thus, we identified time trends in illicit narcotics use within a defined court jurisdiction in a specific risk group to gain knowledge relevant to the involved professionals.

## Methods

### Data Source

Since the early 1990s, the Department of Public Health Medicine (Bavaria, Germany) routinely carries out urine checks for narcotics residues in probationers after a final conviction basing on offences against the German Narcotics Act, as part of their administrative assistance that is provided to the District Court probation service. In 2006, the so-called RUMA^®^ marker system [17] was established to ensure the urine specimens’ validity, providing standardized qualitative biochemical screening results of the collected urine samples without supervision and blinded for all participants in the target group. The dataset used in this study consisted of 2,928 urine samples screened for the detection of one or more substances from 380 probationers who were on probation between January 2006 and December 2019 and screened for the detection of one or more substances: Amphetamines, Opiates, Cocaine, Cannabinoids, Benzodiazepines, Buprenorphine, Barbiturates, LSD, PCP and EDDP.

### Biochemical Analysis

To rule out any sample manipulation, quality assurance measures of the urine samples were performed and included a spit marker analysis for sucrose detection [17, 18], recovery of the polyethylene glycols labeling marker substance, determination of the creatinine content as a marker of urine dilution [17], and a sample check to rule out any sample swapping or adulteration (CEDIA™ DAU Sample Check Assay) [19]. The screening for narcotic residues was carried out by immunoassay (IA) methods for ten relevant substances and substance groups [20]. Positive screening results were confirmed chromatographically with a mass spectrometric detector.

All of the biochemical analysis results were sent to the Department of Public Health Medicine in compliance with all data protection regulations. The data routinely collected throughout the whole observation period was digitized, pooled, and anonymized. The data set was evaluated retrospectively as part of a comprehensive survey.

### Statistical Analysis Methods

The statistical evaluation was carried out with R version 4.2.2. Since a probationer could relapse due to one or more of the nine tested substances, we estimated, using the *cuminc* function of the *tidycmprsk* R package, the cumulative incidence function (CIF) for competing risks data with each substance being a competing risk. The CIF is a proper summary curve for analyzing time to event data in the presence of competing risks [21, 22], because it does not assume independence between events. It also assumes non-informative censoring, that is the censored observations are representative of the population at risk at any time point. The CIF shows for each substance the cumulative relapses over time. It is easily interpretable and allows a direct comparison of the relapse probability between the different substances at given time points, because the cumulative relapse rate for any substances equals the sum of the cumulative relapse rate for each substance. We also estimated the Kaplan-Meier (KM) curve (e.g., 1-CIF), showing the overall survival, that is the probability to stay relapse-free from any substance during the observation period.

The probationers were observed in the study from the first urine screening until the occurrence of the first relapse or until the last follow-up date in the case of none relapse before (= time points of censoring). Because some probationers were observed for 1 day (e.g., had only one urine screening), the observation time of all probationers was shifted by 1 week. The data was right censored, because of probationers who were lost to follow-up or those who did not relapse by the end of their probation period. Loss of follow-up occurred because of a change of address, intercurrent diseases with a need for therapy and intervening judicial convictions for other criminal offences. Gray’s test [23] was used to assess any significant sex and age (categorized) differences in the cumulative relapse rate of each substance. CIF-plots were calculated for significant age and sex differences. The level of significance for all statistical tests was set to 5%.

## Results

### Study Population

The study population included 380 individuals with a median age of 27.5 years (female: 25.9 years *versus* male: 27.7 years), predominantly from 327 male (86.1%) and 53 female probationers. The majority of people within the study cohort belonged to the age groups with the younger participants (until 35 years) and the minority was older than 36 years. The distribution of sex and age of the whole study cohort is summarized in Table 1.

**TABLE 1:** Study population- Age at the first urine screening- and gender-specific stratification of the pooled urine samples (*N* = 2,928) (Relapses in illicit drug use among probationers: Results in a risk group of Public Health Services in Bavaria, Germany, January 2006—December 2019).

Age groups (years)	Sex		Total
	Female	Male	
Younger than 25	18.7% (*n* = 25)	81.3% (*n* = 109)	35.2% (*n* = 134)
26–30	13.9% (*n* = 15)	86.1% (*n* = 93)	28.5% (*n* = 108)
31–35	10.0% (*n* = 6)	90.0% (*n* = 54)	15.8% (*n* = 60)
36–45	6.3% (*n* = 4)	93.7% (*n* = 59)	16.6% (*n* = 63)
Older than 45	20.0% (*n* = 3)	80.0% (*n* = 12)	3.9% (*n* = 15)
Total	13.9% (*N* = 53)	86.1% (*N* = 327)	100.0% (*N* = 380)
Median (Interquartile Range)	25.9 (7.3)	27.7 (10.3)	27.5 (10.2)

*n*, absolute numbers in the subgroups.

*N*, total numbers.

The median time on probation (Interquartile Range = IQR) of the whole cohort was 1 year (IQR = 2), with a minimum period of 1 day and a maximum of 13 years.

### Biochemical Analysis

#### Quality Assurance Measures

The labeling polyethylene glycol marker was recovered in 95.0% (*n* = 2,782) of all urine samples (*N* = 2,928) during the entire observation period (compared to 4.1% and 0.9% with borderline and without marker detection respectively), without any significant age- and sex-specific differences.

In the spit marker test, the sucrose concentration was below 40.0 mg/dL in 93.0% (*n* = 2,723/2,928) and ≥40.0 mg/dL in 7.0% (*n* = 205) of the urine samples.

As for the creatinine level (reference range: ≥0.2 g/L), it was below 0.2 g/L in 6.4% (*n* = 24) and over 0.2 g/L in 93.6% (*n* = 352) of the urine samples among female probationers, and below 0.3 g/L in 8.1% (*n* = 209) and above 0.3 g/L in 91.9% (*n* = 2,356) of the urine samples among male probationers. To detect any urine adulteration, 68.3% of the urine samples (*n* = 2,000) were checked.

#### Time Trends of Illicit Narcotics Use

By far the most urine analyses and most members of the study cohort were carried out for opiates, cannabinoids, amphetamines and cocaine. Of all urine screenings, the greatest relative share of positive detections of narcotics were found for cannabinoids and opiates, followed by amphetamines and cocaine. Further details to the absolute numbers and distributions of the other groups of narcotics are shown in Table 2.

**TABLE 2:** Urine screenings-absolute numbers of substance specific analysis, positive results and numbers of tested persons (Relapses in illicit drug use among probationers: Results in a risk group of Public Health Services in Bavaria, Germany, January 2006—December 2019).

Substance	Total count of screenings	Count of positive screenings	Count of screened subjects	Count of subjects with positive screenings
Opiates	2,866	87	368	61
Cannabinoids	2,848	136	378	86
Amphetamines	2,790	39	363	32
Cocaine	2,586	17	352	16
EDDP (2-ethylidine-1,5-dimethyl-3,3-diphenylpyrrolidine)^a^	755	49	199	31
Buprenorphine^b^	539	11	131	8
Benzodiazepines	517	22	164	13
Barbiturates^c^	318	0	111	0
LSD (Lysergic acid diethylamide)	251	3	78	3
PCP^b^	2	0	2	0

^a^ Not considered in the study because the detection of EDDP, a metabolite of methadone, could not be considered a relapse and subsequently classified as recidivism since all the study subjects were part of an opioid substitution therapy.

^b^ Considered in the study, because none of the probationers was under substitution therapy with this narcotic (=positive urine result = relapse).

^c^ Not considered in the study, because no relapses happened during the whole observation period.

### Relapses Under the Observed Time Period of Probation During 14 Years

**FIGURE 1:**
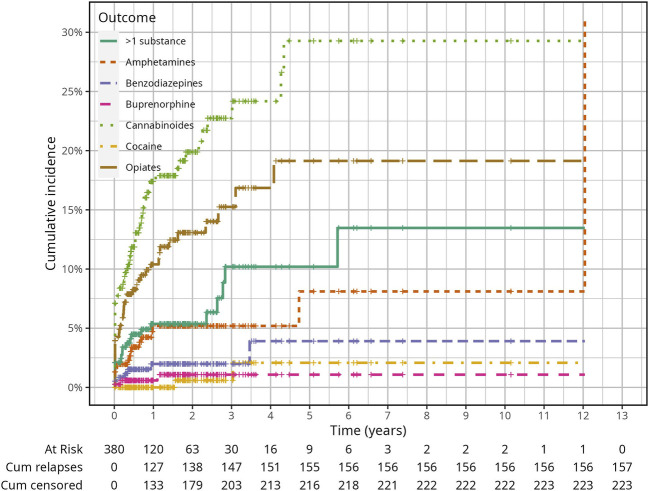
Cumulative Incidence Function of relapsing due to any of all narcotics in the whole study population (Relapses in illicit drug use among probationers: Results in a risk group of Public Health Services in Bavaria, Germany, January 2006—December 2019).

Figure 1 shows the CIF of relapse for each substance in the whole cohort throughout the follow-up period. Remarkably, the cumulative incidence of relapses at one, two and 3 years was the highest for cannabinoids compared to the other substances, with estimates of 17.4% (95% confidence interval = 95% CI: 13.3%–22.0%), 19.9% (95% CI: 15.3%–24.9%), 24.2% (95% CI: 18.2%–30.6%) respectively. The second highest cumulative incidence of relapses occurred with opiates with estimates of 10.4% (95% CI: 7.3%–14.1%), 13.1% (95% CI: 9.4%–17.4%) and 15.2% (95% CI: 10.7%–20.6%) respectively at the same time points. The cumulative incidence of relapses for amphetamines and the concomitant use of more than one substance was comparable until the second observation year with estimates of 5.2% (95% CI: 3.0%–8.3%) and 5.4% (95% CI: 3.2%–8.3%) respectively. At the third year, the cumulative incidence of relapse was higher for the concomitant use of more than one substance than for amphetamines with estimates of 10.2% (95% CI: 5.7%–16.1%) and 5.2% (95% CI: 3.0%–8.3%) respectively. The cumulative incidence of relapses for benzodiazepines was 2% (95% CI: 0.8%–4.1%) and that for buprenorphine was 0.6% (95% CI: 0.1%–2.0%) at 1 year and until the end of the observation period. The cumulative incidence of relapse for cocaine was 0.0%, 0.6% (95% CI: 0.1%–3.0%) and 2.1% (95% CI: 0.3%–7.3%) at one, two and three observation years respectively and no first relapse occurred with LSD until the end of the follow-up period.

**FIGURE 2:**
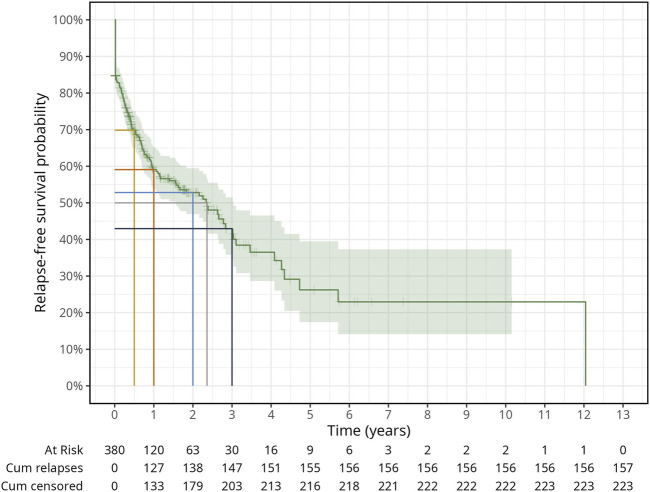
General survival plot for time to relapse with any substance. Tick marks on the survival curve indicate censored subjects (Relapses in illicit drug use among probationers: Results in a risk group of Public Health Services in Bavaria, Germany, January 2006—December 2019).

Figure 2 shows that overall 157 out of 380 probationers relapsed with any substance and 223 were censored because of loss to follow-up (*n* = 57) and staying relapse-free until the end of their probation period (*n* = 166). The 12 years follow-up period ended with a 0.0% relapse-free survival probability because one relapse occurred after the last censoring. The median follow-up time was 0.4 years (IQR = 1.5) overall, 0.7 years (IQR = 1.8) for the censored and 0.2 years (IQR = 0.7) for those who relapsed. Overall, the relapse-free probability since the first urine screening until the end of the probation period was 59% (95% CI: 54%–65%), 53% (95% CI: 47%–59%) and 42% (95% CI: 34%–50%) at the first, second, and third probation years respectively, with a median relapse-free survival time of 2.35 years.

### Age and Sex Differences

We present in Table 3 the numbers of first relapses and the baseline characteristics of the analysis cohort for each competing risk after approximately 12 years of follow-up.

**TABLE 3:** Total number of relapses due to one of the six substances or to concomitant use of more than one substance by age and sex group (Relapses in illicit drug use among probationers: Results in a risk group of Public Health Services in Bavaria, Germany, January 2006—December 2019).

	Total number of relapses per age group (years)					Total number of relapses per sex		Total (%)
Substances	<25	26–30	31–35	36–45	>46	Male	Female	Total
>1 substance	12	4	4	2	0	19	3	22 (14.0)
Amphetamine	3	6	3	2	3	12	5	17 (10.8)
Opiates	8	15	6	12	1	36	6	42 (26.8)
Cannabinoids	25	20	8	8	3	59	5	64 (40.8)
Buprenorphine	1	0	1	0	1	3	0	3 (1.9)
Benzodiazepines	0	3	3	0	1	7	0	7 (4.5)
Cocaine	0	1	0	1	0	2	0	2 (1.3)
Total	49	49	25	25	9	138	19	157 (100.0)

Gray’s test showed statistically significant differences between the age groups and the CIF for the concomitant use of more than one substance (*p*-value = 0.001) and the CIF for cannabinoids (*p*-value = 0.002) respectively and between sexes and the CIF for Amphetamines (*p* = 0.02) (Supplementary Figures S1–S3). Probationers below 25 years of age showed the highest cumulative relapse rate estimates by 1 year for the concomitant use of more than one substance (0.143; 95% CI: 0.07–0.24) and for cannabinoids (0.322; 95% CI: 0.21–0.44) compared to the other age groups (Supplementary Table S1). Female probationers showed a higher cumulative relapse rate estimate by 1 year compared to men (0.139; 95% CI: 0.047–0.28) (Supplementary Table S2). The longest follow-up period was 12.0 and 5.7 years for male and female probationers respectively. We also report in the table the overall cumulative relapse rates by 1 year for all competing events.

## Discussion

Unprecedently, our study estimated the recidivism rate and time trends in the illicit use of specific narcotics in a special target group on probation with a compulsory abstinence order. Under largely standardized conditions and in a strictly circumscribed study area, the vast majority of the defined relapses for the seven considered narcotics groups occurred at the beginning of the probation period along a decrease in the incidence of relapses with time.

### Validity of Pharmacological-Toxicological Urine Analysis

The use of the well validated RUMA^®^ method [19] in conducting the urine drug screening increased to a great extent the informative value of the analysis results, as it allowed the detection of potential manipulation attempts by the target population during sample collection and preservation. In our analyzed samples, we could not ignore the risk of such attempts had occurred, but given its high improbability, we classified it as low. In addition, the biochemical screening’s results are of significant value [24] as they showed little deviations from the methodically defined reference ranges of the specific spit marker, glucose, and creatinine values in the urine samples [19, 25, 26] within our study population.

However, some manipulations, intentional or not, might influence the sample check and the substance-specific detection methods’ results, and as such unknown and unintended distortions had to be classified. For example, the use of eye drops in the cannabinoids detection test can lead to a lower number of positive test results [27]. Several other substances like hair bleach (H_2_O_2_), chromates, soap, vitamin C, detergents, basic and acids solutions can interfere with the drugs screening tests or destroy the drugs themselves [28]. In addition, immunoassay testing has low sensitivity and low specificity due to varying factors, including cross-reactivity with other structurally-similar drugs [29]. For example, we could not rule out the use of psychoactive drugs by our study cohort, which can be given to treat mental illnesses but also can be misused, given the lack of a comprehensive medication and drug history of the study subjects at the time of collection and preservation of the urine samples.

We believe the laboratory findings are reliable and objective and have high internal and external validity given the standardized process of urine sample collection, transport, automated analysis, and data transmission. A methodological strength of our study is that our results largely observed trends of illicit narcotic consumption despite abstinence requirements over a 14 years observation period.

### Addiction, Relapses, and Public Health

Following a thorough literature review, we identified studies reporting only the prevalence of use of single groups of narcotics [1, 12–16], which were not the focus of our recent study. In contrast to our work, these studies did not carry out characteristic time-trend analyses on the use of specific narcotics in the general or the at-risk population, so our results were incomparable to those.

Based on previously reported prevalence of use of some illicit narcotics, we expected cannabinoids and opiates to be the most frequently consumed substances by subjects on probation. Our study aligns with this finding and shows no sex-specific differences, but points out to age-specific differences, with probationers younger than 25 having the highest cumulative relapse rate over their probationary years. Unlike the relapses of cannabinoids and opiates which could be attributed to their highly addictive nature [1, 2, 4, 5]. The most frequent consumption of cannabinoids can be explained by the known consumption trend in the general population, the specific social milieu, and the first use of cannabinoids [30] often by adolescents. In addition, other studies on substance-use disorders and the use of narcotics in subjects on probationary conditions from the Anglo-American region also showed that cannabinoids were the most frequently used substances [29, 31]. That underpins the plausibility of our results in recording recidivism. Yet, the low consumption frequency of LSD was somewhat surprising, possibly indicating underreporting of LSD relapses. That is plausible given that the detection time of LSD in urine is 24 h (it can go up to a few days after the use of this group of substances) and the fact that for some samples the drug control interval was too long [32]. Furthermore, our study showed that female probationers as compared to male probationers, had a higher relapse rate with amphetamines, a finding supported by previous research [33].

Regarding the currently use of almost all routinely tested illicit substance classes, our cohort study showed that the narcotics relapses occurred timely, at the beginning of the observation period for most narcotics by the first probationary year, with substance-specific time intervals. Probationers at the high individual risk of relapse, who violated the substance abstinence order at an early stage of the probation period, were prosecuted for not fulfilling the probation conditions and thus left the study population at that period. At a later stage of the observation period, our study population consisted mainly of probationers with lower relapse risk. More precisely, the probability of relapsing within the first probationary was highest for cannabinoids and opiates, which is not surprising given that opioid use is known to be associated with more intensive relapses [34]. A study from the Anglo-American region, which did not carry out a substance-specific analysis but examined the rate and timing of technical violations by probationers, was able to determine a comparable time-trend of relapse occurrence based on positive drug tests [15].

In addition, the social environment of probationers should be given special attention, given that it can be an important factor for long-term substance abstinence. Late-stage recidivism demonstrated that intensive supervision of individuals at all time points of probation can be a critical factor for abstinence and resocialization of our affected at-risk group. Several risk factors for relapses have been identified including modifiable risk factors such as substance abuse, an antisocial environment, and the need for psychiatric treatment, as well as static, unmodifiable risk factors such as younger age, male gender, and criminal history [6, 35] and especially a female gender for the misuse of amphetamines [33]. Social support is important throughout the recovery process of probationers [7], via connection to others and engagement with recovery-oriented support networks [10, 36]. Further studies investigating the influence of other sociodemographic determinants such as educational qualifications, income, marital status, number of previous convictions, social environment, and the presence of underlying mental illnesses on recidivism during the probation period are needed. Likewise, in the context of probation, the growing importance of use of new and, especially synthetic drugs, may be underestimated, since there are still no reliable tests that can detect the use of such substances [1]. The use of such new synthetic drugs by our study subjects during the probationary period is not recorded nor tested and thus cannot be ruled out. One possibility for future studies would be to conduct brief oral surveys of the probationers at regular intervals–without prosecution–to obtain detailed information about individual substance use. A particular challenge of treating drug addiction continues to be the simultaneous occurrence of mental illness and substance use disorders, which is considered highly problematic since more than 25 years ago [37, 38], as there is still no evidence on the optimal therapeutic management of subjects with those concurrent conditions [38]. When possible, the so called standard therapy and a substitution drug are combined [39]. The substitution treatment for heroin addiction is preferably carried out with methadone and/or buprenorphine [40]. The substitution drugs used have been shown to increase adherence to therapy [39, 40], and reduce illegal drug use [41–43], but their effects are sometimes hampered by the cyclic phases of addiction [44] which is characterized by alternating periods of abstinence and reuse: binge/intoxication, withdrawal/negative effect, and bias/expectation of the substance [45] making recovery from opioid dependence a long-term or lifelong process [46] and according to Caudy et al. [14] requiring an individualized treatment approach and where years after recovery relapses could still occur, indicating that a detox-free period should not be considered as complete recovery. Similarly, a long-term study by Soyka et al. [47] showed that over 75.0% of the patients were still on substitution therapy after 6 years, and only 9.4% of the subjects abstained from drugs during this period.

### Strengths and Limitations

The urine samples were obtained using a marker system, which reliably uncovered, recorded and almost completely ruled out attempts at manipulation of the samples. Due to this standardized process of urine sample collection, transport, automated analysis and data transmission, all laboratory findings were characterized by high internal and external validity, reliability and objectivity. Thus, the results obtained should have largely reflected the real events of illicit drug consumption despite abstinence requirements within a specific risk population, which on the one hand underpins the innovative character of this study and on the other hand can be classified as a methodological strength.

An important limitation of our study was, that our pooled and nearly the complete dataset analyzed reflects only regional and primarily rural conditions due to the small-scale study area and was therefore not representative of the nationwide recidivism of drug users under probation conditions. In addition, due to the pooled dataset, it was not possible to make statements about the recidivism of individual persons, which is to be classified as a weakness of this study and thus requires further corresponding analyses. Nevertheless, the results in question should be characterized by their innovative character, rule out significant selection bias and provide an opportunity for future topic-related studies.

### Conclusion

The low overall rate of positive drug screenings speaks to the effectiveness of probation for people with narcotic offenses in helping them discontinue drug use. The social environment of those affected is often classified as a risk factor for relapse but can also have a supportive effect. Other factors that could influence the recidivism rate during the probation period are worth investigating in future studies: like sociodemographic determinants such as the educational level, income, marital status, number of previous convictions, the presence of underlying mental illnesses and the impact of use of individual substances.
