# Supplementary material for: Relapses in Illicit Drug Use Among Probationers: Results in a Risk Group of Public Health Services in Bavaria
Source: Int J Public Health. 2023 Oct 11;68:1605955. doi: 10.3389/ijph.2023.1605955 (PMC10598279; doi:10.3389/ijph.2023.1605955)
Supplement: Supplementary file 1 [file Table1.pdf]

Supplementary

TABLE S1. Cumulative incidence at 1 year and Gray's test for relapsing due to one of the 6 substances or due to concomitant use of more than one substance by age group (Relapses in illicit drug use among probationers: Results in a risk group of Public Health Services in Bavaria, Germany, January 2006 – December 2019)

			Age group									
			<25		26-30		31-35		36-45		>46	
**Substances**	**Overall cumulative incidence (95% CI)**	**Gray test p.value**	**N**	**Cumulative Incidence (95% CI)**	**N**	**Cumulative Incidence (95% CI)**	**N**	**Cumulative Incidence (95% CI)**	**N**	**Cumulative incidence (95% CI)**	**N**	**Cumulative Incidence (95% CI)**
**>1 substance**	**0.054 (0.032-0.083)**	**0.001*)**	**11**	**0.140 (0.070-0.242)**	**3**	**0.036 (0.009-0.094)**	**2**	**0.030 (0.005-0.093)**	**1**	**0.013 (0.001-0.064)**	**0**	**0.000 (NA-NA)**
Amphetamines	0.052 (0.030-0.083)	0.232	3	0.049 (0.010-0.138)	6	0.072 (0.028-0.142)	2	0.032 (0.006-0.098)	1	0.020 (0.002-0.095)	3	0.174 (0.04-0.386)
Benzodiazepines	0.020F (0.008-0.041)	0.225	0	0.000 (NA-NA)	3	0.034 (0.009-0.089)	2	0.034 (0.006-0.108)	0	0.000 (NA-NA)	1	0.056 (0.003-0.231)
Buprenorphine	0.006 (0.001-0.020)	0.200	1	0.009 (0.001-0.0450)	0	0.000 (NA-NA)	1	0.016 (0.001-0.074)	0	0.000 (NA-NA)	0	0.000 (NA-NA)
**Cannabinoides**	**0.174 (0.133-0.220)**	**0.002*)**	**24**	**0.322 (0.206-0.444)**	**19**	**0.225 (0.141-0.322)**	**6**	**0.091 (0.036-0.177)**	**4**	**0.061 (0.019-0.137)**	**1**	**0.059 (0.003-0.244)**
Cocaine	0.00 (NA-NA)	0.776	0	0.000 (NA-NA)	0	0.000 (NA-NA)	0	0,000 (NA-NA)	0	0.000 (NA-NA)	0	0.000 (NA-NA)
Opiates	0.104 (0.073-0.141)	0.248	7	0.091 (0.036-0.176)	11	0.123 (0.065-0.203)	5	0.080 (0.029-0.164)	10	0.156 (0.078-0.258)	0	0.000 (NA-NA)

*) indicate significant differences at p-value <0.05 (significant differences are in bold); N= absolute numbers of relapses; NA= not available
