# Supplementary material for: Relapses in Illicit Drug Use Among Probationers: Results in a Risk Group of Public Health Services in Bavaria
Source: Int J Public Health. 2023 Oct 11;68:1605955. doi: 10.3389/ijph.2023.1605955 (PMC10598279; doi:10.3389/ijph.2023.1605955)
Supplement: Supplementary file 2 [file Table2.pdf]

Supplementary

TABLE S2. Cumulative incidence at 1 year and Gray's test for relapsing due to one of the 6 substances or due to concomitant use of more than one substance by sex group. (Relapses in illicit drug use among probationers: Results in a risk group of Public Health Services in Bavaria, Germany, January 2006 – December 2019)

		Female		Male	
**Substances**	**Gray test p.value**	**N**	**Cumulative incidence (95% CI)**	**N**	**Cumulative incidence (95% CI)**
>1 substance	0.866	1	0.025 (0.002-0.116)	16	0.058 (0.034-0.091)
**Amphetamines**	**0.020*[)]**	**5**	**0.139 (0.047-0.280)**	**10**	**0.039 (0.02-0.069)**
Benzodiazepines	0.328	0	0.000 (NA-NA)	6	0.023 (0.009-0.047)
Buprenorphine	0.499	0	0.000 (NA-NA)	2	0.007 (0.001-0.023)
Cannabinoides	0.195	5	0.119 (0.041-0.244)	49	0.182 (0.137-0.232)
Cocaine	0.618	0	0.000 (NA-NA)	0	0.000 (NA-NA)
Opiates	0.728	5	0.138 (0.047-0.277)	28	0.099 (0.067-0.138)

*[)]indicate significant differences at p-value <0.05 (significant differences are in bold) ; N= absolute numbers of relapses; NA= not available
