# Supplementary material for: Relapses in Illicit Drug Use Among Probationers: Results in a Risk Group of Public Health Services in Bavaria
Source: Int J Public Health. 2023 Oct 11;68:1605955. doi: 10.3389/ijph.2023.1605955 (PMC10598279; doi:10.3389/ijph.2023.1605955)
Supplement: Supplementary file 3 [file Image2.pdf]

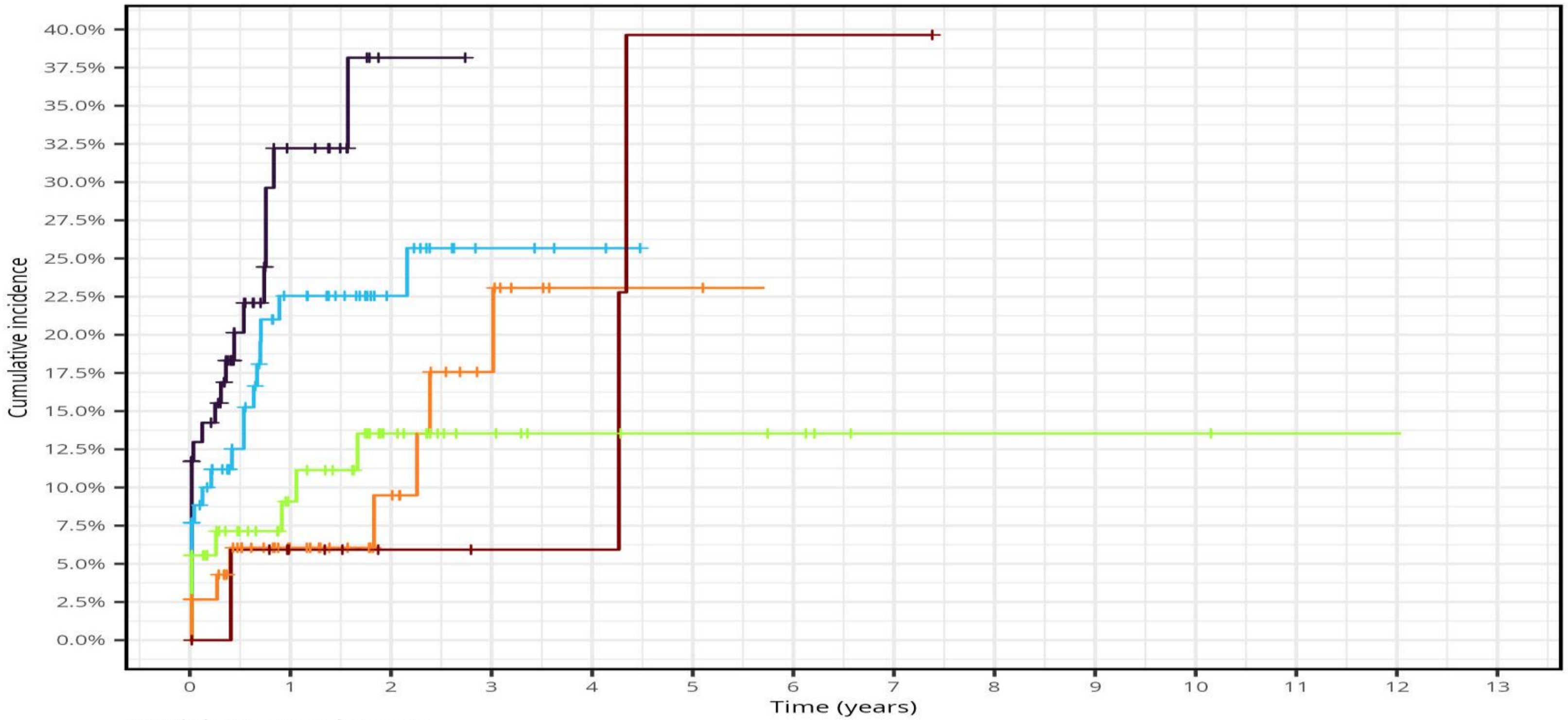

At Risk (Cum relapses)

	0	1	2	3	4	5	6	7	8	9	10	11	12	13
Younger than 25	111 (0)	13 (24)	2 (25)	0 (25)	0 (25)	0 (25)	0 (25)	0 (25)	0 (25)	0 (25)	0 (25)	0 (25)	0 (25)	0 (25)
26 to 30	104 (0)	32 (19)	14 (19)	5 (20)	3 (20)	0 (20)	0 (20)	0 (20)	0 (20)	0 (20)	0 (20)	0 (20)	0 (20)	0 (20)
31 to 35	72 (0)	35 (6)	22 (8)	11 (8)	7 (8)	6 (8)	5 (8)	2 (8)	2 (8)	2 (8)	2 (8)	1 (8)	1 (8)	0 (8)
36 to 45	75 (0)	31 (4)	20 (5)	11 (7)	3 (8)	2 (8)	0 (8)	0 (8)	0 (8)	0 (8)	0 (8)	0 (8)	0 (8)	0 (8)
older than 46	18 (0)	9 (1)	5 (1)	3 (1)	3 (1)	1 (3)	1 (3)	1 (3)	0 (3)	0 (3)	0 (3)	0 (3)	0 (3)	0 (3)

Cum censored

	0	1	2	3	4	5	6	7	8	9	10	11	12	13
Younger than 25	0	52	61	62	62	62	62	62	62	62	62	62	62	62
26 to 30	0	30	44	51	53	55	55	55	55	55	55	55	55	55
31 to 35	0	19	30	38	41	42	43	46	46	46	46	47	47	47
36 to 45	0	28	37	44	49	49	50	50	50	50	50	50	50	50
older than 46	0	4	7	8	8	8	8	8	9	9	9	9	9	9

FIGURE S2. Cumulative incidence function of relapses for cannabinoids by age group. (Bavaria, Germany, January 2006 – December 2019)

Relapses in illicit drug use among probationers: Results of a long term study in a risk group of Public Health Services, Bavaria, Germany, 2006-2019
