# Supplementary material for: Relapses in Illicit Drug Use Among Probationers: Results in a Risk Group of Public Health Services in Bavaria
Source: Int J Public Health. 2023 Oct 11;68:1605955. doi: 10.3389/ijph.2023.1605955 (PMC10598279; doi:10.3389/ijph.2023.1605955)
Supplement: Supplementary file 4 [file Image3.pdf]

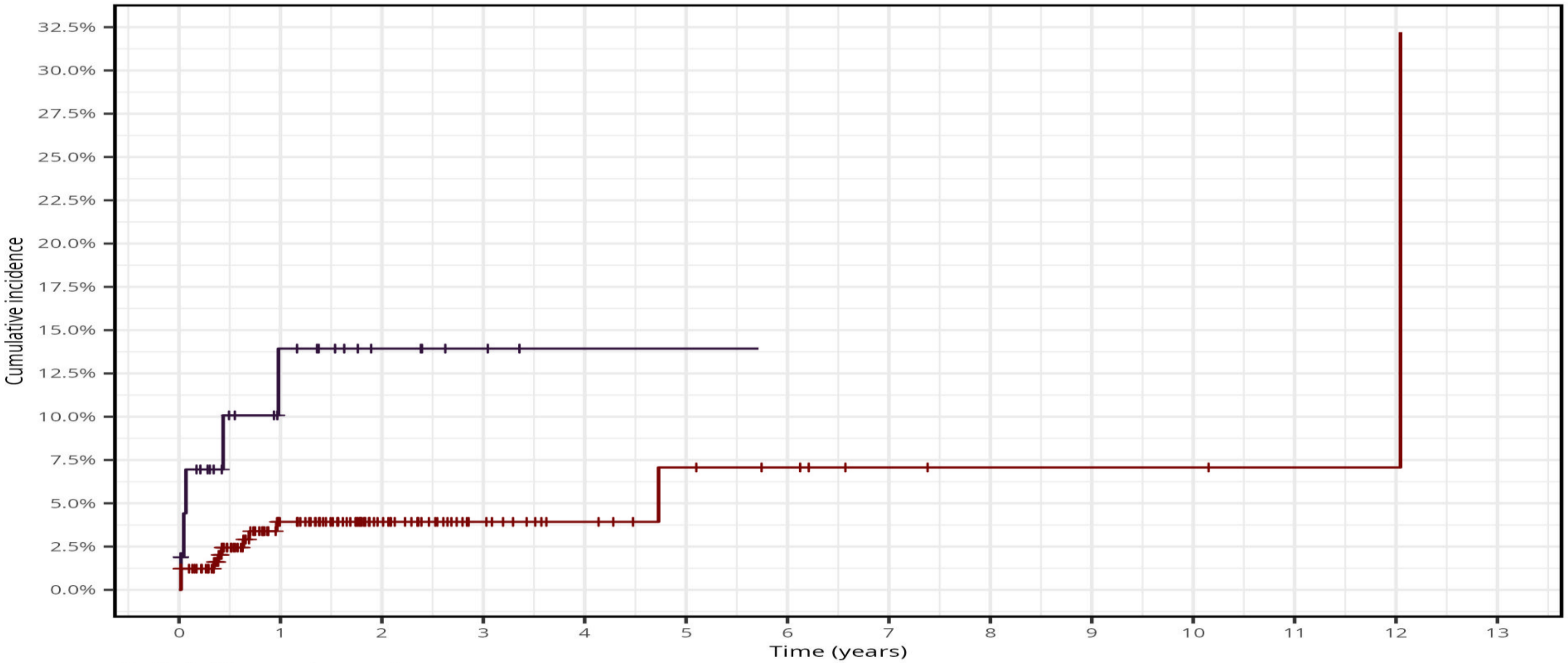

At Risk (Cum relapses)

	0	1	2	3	4	5	6	7	8	9	10	11	12	13
Female	53 (0)	15 (5)	7 (5)	3 (5)	1 (5)	1 (5)	0 (5)	0 (5)	0 (5)	0 (5)	0 (5)	0 (5)	0 (5)	0 (5)
Male	327 (0)	105 (10)	56 (10)	27 (10)	15 (10)	8 (11)	6 (11)	3 (11)	2 (11)	2 (11)	2 (11)	1 (11)	1 (11)	0 (12)

Cum censored

	0	1	2	3	4	5	6	7	8	9	10	11	12	13
Female	0	22	29	32	34	34	34	34	34	34	34	34	34	34
Male	0	111	150	171	179	182	184	187	188	188	188	189	189	189

FIGURE S3. Cumulative incidence function of relapses for Amphetamines by sex (Bavaria, Germany, January 2006 – December 2019)

Relapses in illicit drug use among probationers: Results of a long term study in a risk group of Public Health Services, Bavaria, Germany, 2006-2019
